# Paclitaxel-eluting silicone airway stent with sustained drug release and potent anti-fibrotic activity

**DOI:** 10.3389/fbioe.2026.1805822

**Published:** 2026-05-12

**Authors:** Mingmei Ding, Jiaqi Song, Chen Zhao, Xiyun Yang, Yuehan Ouyang, Zhining Zhuo, Xiaobang Chen, Ruitian Zhu, Xiangqin Wang, Tianhao Yu, Xiumei Tian, Fanwen Yang

**Affiliations:** 1 The Key Laboratory of Advanced Interdisciplinary Studies, The First Affiliated Hospital of Guangzhou Medical University, Guangzhou, China; 2 School of Biomedical Engineering, Guangzhou Medical University, Guangzhou, China; 3 KingMed School of Laboratory Medicine, Guangzhou Medical University, Guangzhou, China; 4 Guangzhou Inspection Testing and Certification Group Co., Ltd., Guangzhou, China; 5 Guangdong Second Provincial General Hospital, Guangzhou, China

**Keywords:** airway stent, anti-fibrosis, drug release kinetics, hydrophilic modification, paclitaxel

## Abstract

**Introduction:**

Benign central airway stenosis (BCAS) remains difficult to manage because restenosis is frequently driven by fibroproliferative responses after intervention. To address this challenge, we developed a long-acting paclitaxel (PTX)-eluting silicone airway stent that integrates hydrophilic modification, a porous drug-reservoir structure, and ultrasound-responsive release.

**Methods:**

Three hydrophilic modifiers (PEG-600, HM-530, and PVP-K17) were screened to improve matrix wettability and drug diffusivity. Coating thickness, pore formation, and cyclic ultrasound stimulation were further optimized. Drug release behavior, release kinetics, and mechanical performance were characterized systematically. In vitro cytocompatibility and anti-fibrotic activity were evaluated using L929, HFL-1, BEAS-2B, and HBE135 cells.

**Results:**

PVP-K17-modified porous stents combined with cyclic ultrasound showed the optimal release profile, achieving 22.85% cumulative PTX release over 90 days while retaining mechanical integrity. Drug release was well fitted by a comprehensive kinetic model (R^2^ > 0.99) incorporating pore-forming (Kt) and ultrasound-enhancement (St) coefficients. Extracts of the modified silicone material met ISO 10993-5 cytocompatibility criteria, and PTX treatment selectively inhibited fibroblasts while showing relatively limited effects on epithelial cell viability.

**Discussion:**

These findings establish an integrated design framework for hydrophilic-modified, ultrasound-activated drug-eluting silicone airway stents that combine sustained local drug delivery, mechanical reliability, and selective anti-fibroproliferative activity.

## 1 Introduction

Central airway obstruction (CAO), arising from benign etiologies such as post-intubation stenosis or malignant diseases, significantly compromises respiratory function and threatens patient survival, thereby necessitating effective clinical intervention. Airway stenting, particularly with silicone-based prostheses, has been widely adopted to maintain luminal patency in cases of complex stenosis or in patients deemed unsuitable for surgical resection. While conventional silicone stents, including the Dumon-type, provide favorable biocompatibility and mechanical support, their long-term efficacy is frequently limited by complications such as granulation tissue hyperplasia, biofilm formation, and stent migration, often mandating stent replacement or removal (Chen et al., 2021; Johnson et al., 2022; Lin et al., 2023; Tian et al., 2022). The development of granulation tissue is primarily triggered by persistent mechanical irritation, foreign body reactions, and a pro-inflammatory microenvironment at the stent–tissue interface. Histological evidence indicates that this process involves excessive fibroblast proliferation, aberrant extracellular matrix (ECM) deposition, and pronounced immune cell infiltration, largely orchestrated by pro-inflammatory cytokines such as IL-1β, IL-6, and TGF-β (Azwal et al., 2022; Henderson et al., 2020; Liu et al., 2021; Motz et al., 2017). Furthermore, epithelial–mesenchymal transition (EMT) and oxidative stress have been identified as key contributors to fibrotic progression, exacerbating airway narrowing and perpetuating the cycle of obstruction and restenosis (Li et al., 2025). Together, these pathophysiological insights underscore the urgent need for functional enhancements to conventional silicone stents—modifications capable of mitigating restenosis and modulating the host tissue response more effectively.

To address the limitations of current stents, particularly restenosis, significant research focuses on improving silicone airway stents through diverse approaches: structural optimization (Jung et al., 2021), material reinforcement (Morad Hasely et al., 2023), anti-protein adhesion coatings (Glumac et al., 2022), antibacterial surfaces (Liu et al., 2020), 3D printing for customized fabrication (Gildea et al., 2018; Schweiger et al., 2018), and critically, the development of drug-eluting stents (DES), which provide superior long-term outcomes relative to bare-metal stents by enabling sustained local drug delivery (Farahmandghavi et al., 2019; Xu et al., 2020). Among therapeutic agents, paclitaxel (PTX) has emerged as a promising candidate owing to its dual anti-fibrotic and anti-inflammatory activity. Preclinical evidence confirms that PTX markedly suppresses both fibroblast proliferation and collagen deposition—two central drivers of restenosis. Consistent with this, PTX-coated balloon catheters and 3D-printed airway stents have demonstrated encouraging results, enhancing airway patency through potent anti-fibrotic effects while preserving epithelial barrier function (Ren et al., 2024; Zhuo et al., 2024).

Despite the therapeutic potential of PTX, achieving controlled and sustained release from silicone-based matrices remains challenging owing to the inherent hydrophobicity of silicone. This characteristic severely restricts water uptake and drug diffusion, typically resulting in an initial burst release followed by a subtherapeutic plateau—a profile inadequate for long-term fibrosis management. Consequently, strategies to modulate the silicone matrix for improved drug release kinetics have become a central research focus. Hydrophilic modification has emerged as a particularly effective approach. Evidence indicates that hydrophilic coatings not only enhance drug elution but also reduce airway tissue damage. For instance, studies involving hydrophilic copolymer coatings have demonstrated their ability to alleviate epithelial injury, minimize mucosal erosion, and suppress inflammatory cell infiltration in preclinical airway models (Cho et al., 2024). These findings align with our previous report, which highlighted the efficacy of Poloxamer 407 in augmenting drug release and extending therapeutic duration (Zhuo et al., 2024).

Polyvinylpyrrolidone (PVP)—a hydrophilic polymer with a well-established safety record—has attracted sustained interest for its excellent biocompatibility, film-forming capability, and capacity to modulate drug-release kinetics. Widely used in biomedical materials, including wound dressings and drug-delivery systems (Ge et al., 2023; Gheorghita et al., 2022; Teodorescu et al., 2019; Thongsuksaengcharoen et al., 2020; Tsatsakis et al., 2019; Wu et al., 2022; Zhang et al., 2014), PVP improves surface wettability and water uptake in silicone-based matrices, thereby smoothing early burst and extending release to support sustained local therapy. Consistent with this behavior, Tang et al. reported favorable safety and lubricity of PVP hydrophilic coatings on ureteral stents (Tang et al., 2024).

Beyond chemical approaches, physical modification—particularly pore formation—has been leveraged to optimize drug release from silicone stents. Introducing a porous architecture increases the effective surface area and shortens diffusion paths, thereby improving drug permeability and homogenizing release profiles (Ahuja and Pathak, 2009; Chen et al., 2024). Consistent with these mechanisms, recent *in vivo* and histological evaluations of fenestrated silicone stents have shown more effective granulation-tissue reduction and improved local tissue integration compared with conventional designs, findings that indirectly support enhanced drug diffusion and mechanical compliance of porous stents in clinical applications (Wang et al., 2025). Furthermore, ultrasound-mediated drug activation has emerged as a promising strategy for enhancing drug diffusivity and matrix permeability. Through acoustic cavitation and microstreaming effects, ultrasound energy can disrupt drug-matrix binding, effectively simulating the dynamic pressure fluctuations characteristic of coughing in stenotic airways (Díaz-Jimenez and Rodriguez, 2023; Tao et al., 2025; Wu and Nyborg, 2008; Zhang et al., 2022).

In this study, we designed a PTX-eluting silicone airway stent with a multi-layered drug-reservoir structure, engineered to achieve sustained local drug delivery in benign airway stenosis. To optimize hydrophilicity and regulate release kinetics, three distinct hydrophilic modifiers—PEG-600, HM-530, and PVP-K17—were systematically incorporated into the silicone matrix. We comprehensively evaluated the effects of hydrophilic modification, porous architecture, and ultrasound stimulation on drug release behavior. Multiple drug release models—including zero-order, first-order, Higuchi, and a refined comprehensive model—were developed and validated to characterize underlying release mechanisms across varying processing conditions. Additionally, a novel dual-factor release model incorporating both pore-forming (K_t_) and ultrasound-enhancement (S_t_) coefficients was established, enabling accurate prediction of multi-phase drug elution profiles. Cytocompatibility was confirmed in human bronchial epithelial cells (HBE135 and BEAS-2B), while the anti-fibrotic activity of released PTX was demonstrated in human lung fibroblasts (HFL-1), supporting its selective therapeutic efficacy against fibrosis.

## 2 Materials and methods

### 2.1 Materials

The two-component silicone elastomer (KEG-2000-70A/B) was obtained from Xinyue Organic Silicon International Trade (Shanghai) Co., Ltd. PEG-600, HM-530, PVP-K17, and PBS buffer powder were sourced from Tianjin Damao Chemical Reagent Factory, Guangzhou Haierma Vegetable Oil Co., Ltd., Shenzhen Wenle Biotechnology Co., Ltd., and Guangzhou Jiebeisi Technology Co., Ltd., respectively. Pharmaceutical-grade paclitaxel (PTX), sodium dodecyl sulfate (SDS), acetonitrile, dimethyl sulfoxide (DMSO), and ethyl acetate were supplied by Aladdin Reagent (Shanghai) Co., Ltd. BEAS-2B, HBE135, L929, and HFL-1 cell lines were procured from Wuhan Pricella Biotechnology Co., Ltd. (China). MTT cell proliferation and cytotoxicity assay kits, reactive oxygen species (ROS) detection kits, and Calcein AM/PI double-stain live/dead assay kits were provided by Beyotime Biotechnology Co., Ltd. and Elabscience Biotechnology Co., Ltd. (China).

### 2.2 Sample preparation

#### 2.2.1 Preparation of silicone sheets loaded with PTX

PTX was dissolved in ethyl acetate at a weight ratio of 1:9 (PTX to solvent) to form a homogeneous drug solution. KEG-2000-70A silicone base was then incorporated into the PTX solution and stirred electromagnetically at 300 rpm for 5 min. Subsequently, KEG-2000-70B crosslinker was added, followed by additional stirring at 300 rpm for 5 min to ensure complete mixing. The resulting mixture was transferred into a square plate mold. To fabricate PTX-loaded silicone sheets, the cast samples were first air-dried under ventilation for 2 h to allow ethyl acetate evaporation, and then thermally cured in an oven at 80 °C for 60 min. After naturally cooling to room temperature over 24 h, the cured sheets were cut into 1 cm × 1 cm films with controlled thicknesses of 0.05, 0.10, 0.20, 0.30, and 0.40 mm.

#### 2.2.2 Preparation of silicone/PEG sheets loaded with PTX

PTX was first blended with PEG-600 at a weight ratio of 1:2.5 to form a uniform PTX/PEG-600 mixture. To systematically evaluate the influence of hydrophilic modification on drug release behavior, formulations with varying PEG-600 contents—0%, 2.5%, 5%, 7.5%, and 12.5%—were prepared. The KEG-2000-70A silicone base was introduced into the PTX/PEG-600 mixture and magnetically stirred at 300 rpm for 5 min. KEG-2000-70B crosslinking agent was then added, and stirring continued under identical conditions for an additional 5 min. The homogenized mixture was cast into a square mold and thermally cured in an oven at 80 °C for 60 min. After cooling naturally for 24 h, the resulting samples were cut into 1 cm × 1 cm sheets with a consistent thickness of 0.1 mm.

#### 2.2.3 Fabrication of the drug-loaded stent

**SCHEME 1:**
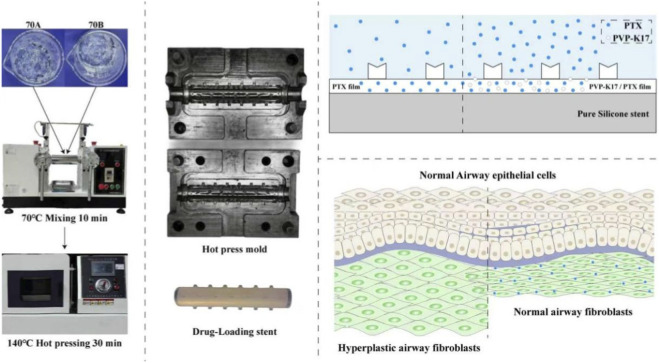
Fabrication and Drug Release Mechanism of the Paclitaxel Airway Stent.

The two-component silicone system (KEG-2000-70A/B) was initially compounded using a two-roll mill at 70 °C for 10 min to achieve a homogeneous silicone matrix. The pre-mixed silicone was uniformly spread into the PTFE mold and hot-pressed at 100 °C for 2–3 min, yielding a slightly adhesive, semi-solid preform that served as the stent’s structural base layer. The fabrication process and proposed drug release mechanism of the paclitaxel airway stent are illustrated in Scheme 1.

In parallel, a drug-loading solution was prepared by dissolving PTX in ethyl acetate. To fabricate the drug-eluting coating, 5 wt% of each hydrophilic modifier—PEG-600, HM-530, and PVP-K17—was individually blended with KEG-2000-70A and KEG-2000-70B. Each mixture was stirred at 70 °C for 10 min to achieve homogeneous modification. The PTX solution was then introduced dropwise into the modified silicone under continuous stirring at 70 °C for an additional 10 min. The drug-loading modified silicone was cast into a custom mold and hot-pressed at 80 °C for 60 min to form drug-loaded film.

During final assembly, the drug-loaded film was placed flat, and the preformed base layer was accurately positioned over it. The bilayer structure was carefully aligned and wrapped around a metal core rod, ensuring the drug-loaded layer remained outermost. The assembly was then placed into a hot-pressing mold and compression-molded at 100 °C for 25 min. After hot pressing, the mold was transferred to a cooling station and allowed to equilibrate for 10 min. Upon cooling, the mold was opened and the finished stent was gently demolded from the metal core, resulting in a fully constructed drug-eluting multilayer silicone airway stent.

### 2.3 Characterizations

#### 2.3.1 Water bath simulation experiment

To simulate the pore-forming process resulting from PEG dissolution within the silicone matrix, sheet samples (10 mm × 10 mm) were immersed in distilled water, heated to boiling, and maintained at this temperature for varying durations. The resulting microstructural changes were then characterized by scanning electron microscopy (SEM).

#### 2.3.2 Micromorphological structure

The sheet samples were sputter-coated with a thin gold layer and subsequently examined under a scanning electron microscope (SEM; model MVE 0329591690, PHENOM, Shanghai, China) at varying magnifications to characterize their surface morphology.

#### 2.3.3 Standard curve of drug release

Paclitaxel (PTX, 2.5 mg) was accurately weighed and dissolved in a 60% methanol/acetonitrile mixture in a 25 mL volumetric flask to prepare a stock solution with a concentration of 100 μg/mL. This solution was then serially diluted to obtain standard solutions at concentrations of 0.5, 1.0, 5.0, 10.0, and 50.0 μg/mL. Chromatographic analysis was performed using a High Performance Liquid Chromatography system (HPLC 1290 Infinity II, Agilent, USA) under the following conditions: injection volume 20 μL, mobile phase acetonitrile/water (60:40, v/v), flow rate 1 mL/min, column temperature 25 °C, Agilent Eclipse Plus C18 column (4.6 × 250 mm, 5 μm), and detection wavelength 227 nm. A standard calibration curve was generated by linear regression of peak area against drug concentration.

#### 2.3.4 Release behavior of drug-loaded sheets

The drug-loaded sheet was put into 50 mL centrifuge tube, added with 40 mL of PBS (pH 7.4) containing 1% SDS (Shen et al., 2013) as the extraction solvent, and then subjected to extraction in a water bath thermostatic shaker (THZ-92A, BOXUN, SH, China) at 37 °C and 70 r/min shaking frequency for 2 days. After 2 days of extraction, the drug release solution was taken out, a new extraction solvent was added, and so on. The concentration of the drug release solution was quantified by HPLC.

Pore-making assisted release was achieved by puncturing the stent surface with needles (inner diameter: 0.20 mm, spaced 1.0 mm apart) to a depth of 0.20 mm, thereby constructing microporous channels to enhance the specific surface area for drug release.

Ultrasonic-assisted release was performed by immersing the stent in the release medium and subjecting it to ultrasound treatment (frequency: 40 kHz, power: 180 W, duration: 15 min) every 24 h.

#### 2.3.5 Drug release kinetics

The drug release profiles of various paclitaxel-loaded airway stents were evaluated using multiple kinetic models, including zero-order, first-order, Higuchi, Ritger–Peppas, diffusion-relaxation, and a comprehensive model. To better represent the complexity of stent-based drug delivery, a multifactorial integrated release model was developed that incorporated key influencing factors such as coating thickness, hydrophilic modification, porosity, and ultrasound-assisted stimulation. The fitting performance of each model was quantitatively assessed using the adjusted coefficient of determination (*R* ^2^), residual sum of squares, and chi-square reduction. These statistical metrics collectively provided a rigorous basis for identifying the most suitable kinetic model to characterize the *in vitro* release behavior of paclitaxel.

#### 2.3.6 *In vitro* biocompatibility of the stent extracts

##### 2.3.6.1 Cell viability

Sample preparation for the material extract followed ISO 10993–12 guidelines. Stent materials were extracted in complete medium at a ratio of 0.2 g/mL using a constant-temperature shaker at 37 °C for 24 h to obtain 100% extract. In addition, to quantitatively evaluate the biological effect of paclitaxel under release-relevant conditions, a PTX treatment medium was prepared in the extract medium of the drug-free PVP-K17-modified silicone material, based on the average PTX release concentration during the first 10 days of the optimized stent release profile (9.3 μg/mL).

Cell viability was evaluated using an MTT assay kit (Beyotime, China). L929 and HFL-1 cells were used as fibroblast models, whereas BEAS-2B and HBE135 cells were used as epithelial cell models. Cells were seeded in 96-well plates at 1 × 10^4^ cells/well and divided into four groups: NC (complete medium), PVP-K17 group (100% extract of the drug-free PVP-K17-modified silicone material), PTX group (drug-free PVP-K17-modified silicone extract containing 9.3 μg/mL paclitaxel), and PC (positive control). Cell viability was assessed after 1, 3, and 5 days of treatment.

At each time point, MTT solution was added to each well and incubated for 4 h at 37 °C. The resulting formazan crystals were dissolved in 100 μL of formazan solvent, and absorbance was measured at 570 nm (primary wavelength) and 650 nm (reference wavelength) using a microplate reader (Spark 10M, TECAN, Germany). Cell viability was normalized to the NC group. In accordance with ISO 10993–5, materials resulting in cell viability of ≥70% relative to the negative control were considered non-cytotoxic.

##### 2.3.6.2 Live-dead staining

Cells were seeded in six-well plates at ≥ 4 × 10^4^ cells/well and treated according to the viability assay protocol. After 24 h, live and dead cells were stained using a calcein-AM/PI double-staining kit by applying 500 μL of staining solution per well and incubating for 30 min. Images were acquired with a Zeiss inverted fluorescence microscope (Axio Observer 5, ZEISS, Germany) under bright-field and fluorescence modes (excitation: 417 nm and 517 nm) to enhance staining clarity.

##### 2.3.6.3 Reactive oxygen species (ROS) assay

HBE135 and HFL-1 cells were cultured in 12-well plates at ≥ 3 × 10^5^ cells/well. In addition to the viability assay, a parallel set of wells was prepared for ROS detection, with a ROS positive control reagent applied at a final concentration of 50 μg/mL as directed by the assay kit. After 24 h of treatment, cells were incubated with 300 μL of ROS detection reagent (Beyotime, China) for 20 min. Fluorescence imaging was performed using the Zeiss inverted microscope (Axio Observer 5, Zeiss, Germany) under bright-field illumination and 488 nm excitation.

#### 2.3.7 Statistical analysis

All data were derived from three independent experimental replicates and are expressed as mean ± standard deviation (SD). Statistical comparisons were performed by two-way analysis of variance (ANOVA), with a significance level defined as α = 0.05. A P-value ≤0.05 was considered statistically significant.

## 3 Results

In clinical practice, airway stenting remains an important interventional approach for managing benign central airway stenosis (BCAS). However, its long-term efficacy is often compromised by recurrent restenosis driven by chronic inflammation and fibroproliferative responses following stent implantation. Local drug delivery *via* stents represents a promising strategy to address this challenge, offering site-specific therapy with high local drug concentrations, minimized systemic exposure, and enhanced treatment efficiency. Conventional drug-eluting coatings—typically only a few micrometers thick—are limited by low drug loading capacity and short release duration, making them unsuitable for long-term airway management. To overcome this constraint, embedding therapeutic agents within the bulk matrix of silicone stents has been proposed as an effective method to increase drug payload. Yet, the inherent hydrophobicity of silicone restricts drug diffusion, resulting in lower release rate and inadequate cumulative release.

To address these limitations, this study introduces hydrophilic modification as a means to modulate the physicochemical properties of silicone-based stent matrices. We systematically evaluated the mechanical properties and drug release behavior of paclitaxel-loaded silicone systems under varying hydrophilic conditions. Moreover, engineered porous microstructures and ultrasound-assisted stimulation were incorporated as externally applied physical strategies to modulate drug release behavior. A comprehensive set of cytocompatibility assays using human bronchial epithelial cells (BEAS-2B and HBE135) and human lung fibroblasts (HFL-1) was conducted to assess both biosafety and functional selectivity, providing critical insights into the therapeutic potential of paclitaxel in preventing airway restenosis.

### 3.1 Effect of film thickness on drug release

**FIGURE 1:**
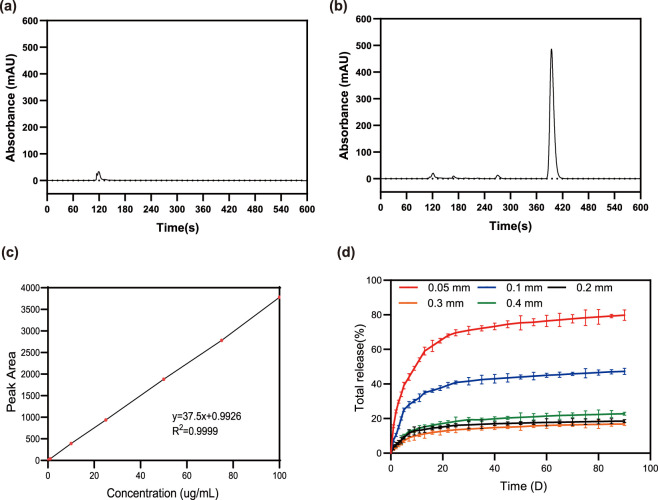
Solubility, quantification, and release behavior of PTX from silicone films. **(a)** UHPLC chromatogram of PTX in PBS (pH 7.4), showing a weak peak at ∼6.5 min, indicating limited aqueous solubility. **(b)** UHPLC chromatogram of PTX in SDS-supplemented PBS, with a significantly enhanced peak at ∼6.5 min, demonstrating improved solubilization. **(c)** Calibration curve of PTX (concentration: 0.1–100 μg mL^-1^; peak area) fitted by y = 37.5x + 0.9926 (*R* ^2^ = 0.9999). **(d)** Influence of film thickness on PTX release from PS films in SDS-containing PBS.

The solubility of PTX in phosphate-buffered saline (PBS) was initially assessed by ultra-high-performance liquid chromatography (UHPLC). As shown in Figure 1a, the chromatogram of PTX in PBS (pH 7.4) displayed only a faint peak at approximately 6.5 min, indicating its limited solubility in aqueous medium. In contrast, when 1% sodium dodecyl sulfate (SDS) was added to the PBS (Figure 1b), the PTX peak intensity increased markedly, demonstrating the solubilizing effect of SDS. This enhancement can be attributed to the micelle-forming capability of SDS, which improves the dissolution of hydrophobic drugs such as PTX (Choi et al., 2019; Saha et al., 2023). Consistent with this, prior studies have confirmed that the addition of 1% (w/v) SDS to PBS (pH 7.4) significantly increases PTX solubility, thereby facilitating reliable HPLC quantification (Shen et al., 2013).

A standard calibration curve was constructed using PTX solutions with known concentrations ranging from 0.1 to 100 μg/mL. As illustrated in Figure 1c, the peak area showed a strong linear correlation with PTX concentration over this range (y = 37.5x + 0.9926, *R* ^2^ = 0.9999), confirming the high accuracy and reproducibility of the UHPLC method for PTX quantification. This calibration relationship served as a reliable basis for determining cumulative PTX release from drug-loaded films in subsequent release studies.

The release profiles of PTX from pure silicone (PS) films (Figure 1d) exhibited biphasic kinetics, characterized by an initial burst release followed by a sustained, slower release phase. This pattern is consistent with typical drug release behavior from polymeric matrices, where rapid initial diffusion is followed by a declining release rate as the system approaches equilibrium. Notably, 0.05 mm thick films showed the most rapid release, with 79.8% of PTX released within 90 days. In comparison, 0.10 mm films released 47.3% over the same period, while films with thicknesses ≥0.20 mm exhibited substantially slower release, with cumulative amounts below 25% after 90 days.

These results demonstrate that drug release kinetics can be effectively modulated by varying film thickness. Thin films (e.g., 0.05 mm) enable short-term, rapid drug delivery, whereas thicker films (≥0.20 mm), due to their increased diffusional path length, provide prolonged release suitable for long-term therapy. Owing to the structural fragility and handling challenges associated with the 0.05 mm films, the 0.10 mm thickness was selected for all subsequent experiments.

### 3.2 Effect of PEG-600 modification on PTX release

In previous experiments, 0.10 mm-thick PTX-loaded silicone films demonstrated limited release performance, with only 47.3% cumulative release over 90 days. To enhance drug release, the silicone matrix was modified with PEG-600—a low-molecular-weight, water-soluble polymer commonly used in pharmaceutical formulations as a plasticizer, co-solvent, and solubilizing agent (Gullapalli and Mazzitelli, 2015). PEG-600 has been shown to improve the dissolution and diffusivity of poorly soluble drugs by increasing the water permeability of polymeric matrices.

**FIGURE 2:**
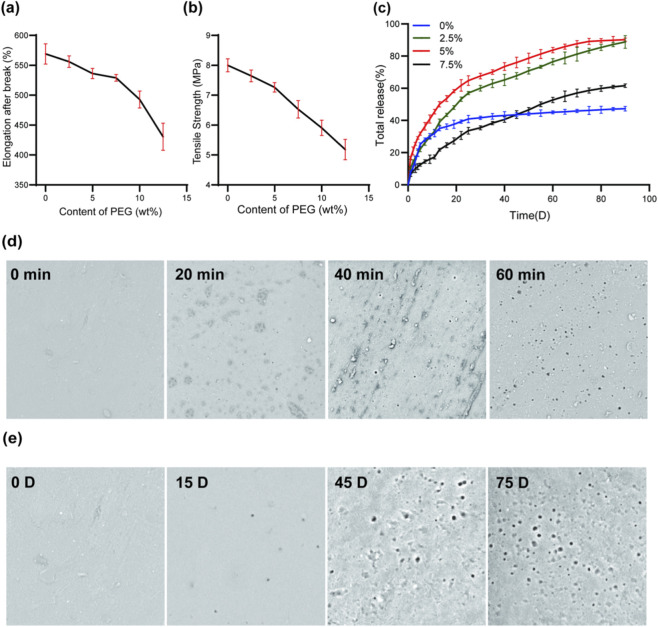
PEG-600 modification of silicone films and its effects on mechanical properties, microstructure, and drug release behavior. **(a)** Elongation at break of films as a function of PEG-600 content (0–12.5 wt%). **(b)** Tensile strength of films *versus* PEG-600 loading. **(c)** Cumulative PTX release profiles from films with different PEG-600 contents (0–7.5 wt%) in SDS-containing PBS over time. **(d)** SEM image of film with 5 wt% PEG-600 after water bath treatment. **(e)** Surface morphology of drug-loaded films (5 wt% PEG-600) after different release durations, where dark pores indicate voids formed following PTX elution.

Mechanical testing (Figures 2a,b) indicated that increasing the PEG-600 content progressively reduced both tensile strength and elongation at break. PS films exhibited a tensile strength of 8.0 MPa and elongation at break of 569.0%, whereas films containing 12.5% PEG-600 showed reduced values of 5.2 MPa and 430.6%, respectively. This decline in mechanical properties suggests that PEG-600 incorporation partially disrupts the cross-linked silicone network, introducing hydrophilic domains that weaken the structural integrity of the matrix. Nevertheless, such structural loosening may facilitate drug release.

As shown in Figure 2c, PEG-600 modification markedly enhanced PTX release. Films with 5% PEG-600 achieved the highest cumulative release (90.2%) after 90 days, followed by those with 2.5% PEG-600 (88.8%). These values represent nearly double the release efficiency of unmodified silicone. However, further increases in PEG-600 content (e.g., 7.5% and 12.5%) led to a decline in drug release (61.6% or lower), likely due to excessive plasticization or phase separation, which may compromise pore connectivity or matrix stability and hinder drug diffusion.

SEM analysis (Figure 2d) of 5% PEG-600 films after water bath treatment revealed time-dependent micropore formation resulting from PEG-600 dissolution. Initially, PEG was uniformly dispersed as isolated hydrophilic domains within the matrix. Upon water immersion, PEG leached out, generating interconnected pore structures that increased in number and uniformity over time. These micropores served as effective diffusion pathways for drug release. Further morphological observations (Figure 2e) confirmed that PTX release was associated with increasing pore size and surface roughness. Before release, drug crystals were visible on a smooth film surface. As incubation proceeded, the crystals diminished while pores expanded, illustrating the dynamic relationship between hydrophilic domain leaching and drug diffusion.

Collectively, these findings indicate that PEG-600 enhances paclitaxel release through a dual mechanism: it improves matrix water permeability and promotes *in situ* pore formation *via* leaching, synergistically accelerating drug diffusion. However, an optimal concentration range exists, as excessive PEG-600 compromises both matrix properties and release performance, highlighting the importance of balanced formulation design for sustained drug delivery applications. Supplementary FTIR and TGA analyses of PEG600-containing silicone samples provided supportive evidence that the system retained basic bulk physicochemical compatibility, without obvious chemical structure disruption or marked thermal instability (Supplementary Figures. S1c, d, left panels). Nevertheless, because these methods do not directly resolve local phase organization, the possibility that excessive PEG600 loading promotes microphase separation within the silicone matrix cannot be fully excluded.

### 3.3 Design and release profiles of airway stents

**FIGURE 3:**
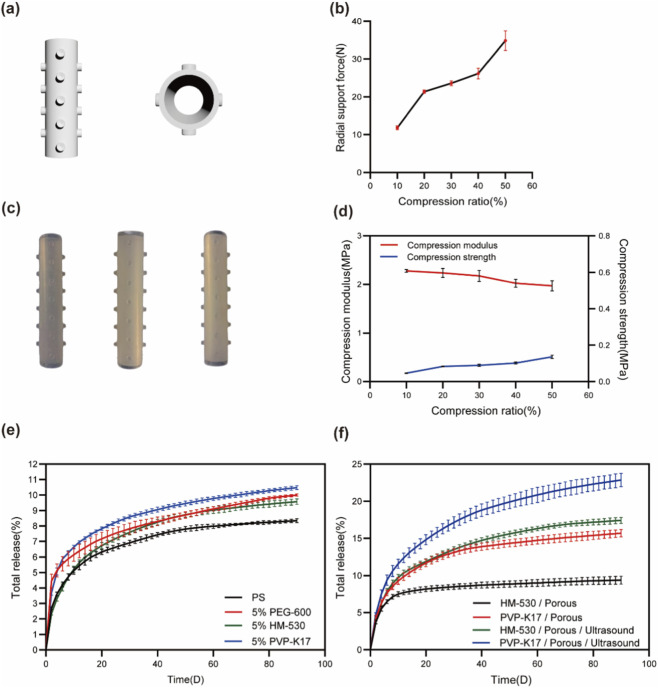
Characterization and drug release profiles of airway stents. **(a)** 3D model of the stent structure. **(b)** Radial support force as a function of compression ratio. **(c)** Photographs of fabricated stents with different hydrophilic modifications (left: 5% PEG-600; middle: 5% HM-530; right: 5% PVP-K17). **(d)** Compression modulus and Compression strength as a function of compression ratio. **(e)** Cumulative drug release from stents with different hydrophilic modifiers. **(f)** Cumulative drug release from stents subjected to different processing strategies.

The three-dimensional model of the drug-eluting airway stent was designed in 3ds Max software, as shown in Figure 3a. The stent exhibits a bi-layered tubular structure with an inner radius of 6.5 mm, an outer radius of 8 mm, and a wall thickness of 1.5 mm, yielding a total length of 80 mm. To improve positional stability and migration resistance, cylindrical protrusions (radius: 1.5 mm; height: 2.4 mm) were symmetrically distributed along the outer surface of the inner layer, comprising seven anteroposterior and six lateral rows. Each protrusion is designed with a central concavity to enhance mucosal adhesion and anchoring capability.

Photographic documentation of the fabricated stents (Figure 3c) revealed uniform morphology with smooth drug-eluting surfaces, confirming the structural consistency and manufacturing reliability of the hot-press molding process. Mechanical analysis (Figures 3b,d) indicated a progressive enhancement in radial support force and compressive strength under increasing radial strain, while the compressive modulus exhibited a corresponding decline. Under 30% and 50% radial compression, the stents achieved radial support forces of 23.63 N and 34.86 N, with compressive strengths of 0.33 MPa and 0.51 MPa, respectively. These mechanical characteristics indicate that the stent possesses a mechanically relevant basis for further translational evaluation in airway applications.

Analysis of cumulative drug release profiles (Figure 3e) demonstrated that the PS stent exhibited an initial burst release, with 2.69% of PTX eluted within the first 48 h. The release rate then decreased markedly to 0.16% by day 20, stabilizing between days 70 and 90 (Supplementary Table S1). The total cumulative release over 90 days reached only 8.34%, significantly lower than the 47.3% observed for standalone drug-loaded silicone films. This pronounced discrepancy stems from the stent’s structural design: the integration of the drug-loaded layer with the stent base layer reduces the contact area with the extraction medium by approximately half, thereby decreasing the effective surface area available for drug diffusion.

To overcome this limitation, the drug-loaded layer was modified with hydrophilic agents PEG-600, HM-530, and PVP-K17. As shown in Figure 3c, all modified stents displayed uniform surface morphology and preserved structural integrity, indicating consistent manufacturing quality. Supplementary segment-based release testing further showed that PTX release behavior was reasonably consistent along both the longitudinal and circumferential directions of the bilayer stent, thereby strengthening confidence in dose uniformity and local delivery consistency (Supplementary Figures. S1f, g). The cumulative release profiles over 90 days are presented in Figure 3e, with detailed kinetic data available in Supplementary Table. S1.

During the early release phase (days 0–10), the PEG-600–modified stent exhibited the highest release rate, attributable to its pronounced hydrophilicity and rapid pore-forming ability upon hydration. In the intermediate phase (days 10–40), the PVP-K17-modified stent demonstrated more sustained release behavior compared to the other formulations. By the late phase (days 40–90), the release rates decreased markedly in all groups, with negligible differences among them. The release pattern is governed by the dynamic evolution of the drug concentration gradient within the stent matrix. The initially high paclitaxel concentration facilitates rapid diffusion, which gradually slows as the concentration gradient declines—a behavior consistent with Fickian diffusion mechanisms. After 90 days, the PVP-K17-modified stent achieved the highest cumulative drug release (10.48%), followed by PEG-600 (10.00%) and HM-530 (9.57%), all of which exceeded the unmodified stent (8.34%). These findings verify that hydrophilic modification enhances drug release efficiency by improving matrix wettability and inducing microstructural porosity.

In summary, the incorporation of hydrophilic modifiers into the stents significantly enhanced paclitaxel release performance without compromising mechanical stability, highlighting a practical approach to achieving sustained local drug delivery in airway stent applications.

### 3.4 Stent processing techniques and drug release

As shown in Figure 3e, hydrophilic modification of the stents significantly enhanced cumulative drug release compared to the PS stent, confirming its role in promoting drug elution. However, the initial burst release observed with PEG-600 modification is suboptimal for airway stents, which require sustained and stable long-term release. Therefore, PVP-K17- and HM-530-modified stents were selected for further investigation. Common strategies to enhance drug release—such as modulating temperature, pH, film thickness, introducing micropores, or applying external energy (Zheng et al., 2025)—were considered. Given the physiological stability of temperature and pH *in vivo* and the practical challenges of reducing film thickness, pore formation and ultrasound assistance were selected as the most viable approaches.

To evaluate the effect of pore formation on drug release, PVP-K17- and HM-530-modified stents were subjected to micro-perforation. The 90-day cumulative release profiles are shown in Figure 3f, with comparative data before and after pore formation provided in Supplementary Table S2. The HM-530/Porous stent exhibited a cumulative release of 9.39%, similar to its non-porous counterpart. In contrast, the PVP-K17/Porous stent showed a marked 49.6% increase in cumulative release (from 10.48% to 15.68%), indicating that pore formation substantially enhanced drug elution in this formulation. This improvement aligns with Fick’s law, as increased specific surface area and diffusion coefficient facilitate drug release.

Given the limited release enhancement from pore formation alone, ultrasound-assisted release was further applied to HM-530/Porous and PVP-K17/Porous stents. The 90-day cumulative release results are shown in Figure 3f, with detailed comparisons in Supplementary Table S3. The PVP-K17/Porous/Ultrasound stent achieved a cumulative release of 22.85%, and the HM-530/Porous/Ultrasound stent reached 17.42%, representing increases of 45.7% and 85.5%, respectively, over their non-ultrasound counterparts. These results demonstrate the pronounced effect of ultrasound in enhancing drug release from modified stents.

As summarized in Supplementary Table S2, ultrasound significantly boosted release rates for both stent types. On day 10, the HM-530-modified stent showed a 130% increase in release rate, while the PVP-K17 stent increased by 36.9%. Improvements were also observed on days 30 and 60. Notably, the PVP-K17 stent maintained a measurable release rate (0.11%) even on day 90, suggesting continued drug elution.

The enhanced cumulative release under ultrasound is likely attributable to cavitation effects induced by cyclic ultrasonic vibrations. Microbubbles generated within the drug-loaded matrix undergo repeated expansion and contraction, producing fluid shear stresses that disrupt the drug–matrix interface, transiently increasing porosity and membrane permeability—a phenomenon well-documented in reviews on ultrasound-mediated drug delivery (Tao et al., 2025). Additionally, ultrasonic energy may impart mechanical shear and tensile stresses that weaken intermolecular interactions (e.g., van der Waals forces or hydrogen bonding) between PTX and the silicone matrix, thereby facilitating drug desorption and diffusion (Wei et al., 2021). Together, cavitation-induced permeability enhancement and disruption of drug–polymer interactions contribute to the observed increase in instantaneous and cumulative drug release under ultrasound.

In summary, the optimal formulation and processing strategy for the paclitaxel-loaded airway stent comprised: a 0.1 mm drug-loaded film thickness, incorporation of 5% PVP-K17 as hydrophilic modifier, a drug loading of 24 mg, and the combined application of pore formation and ultrasound assistance, achieving a 90-day cumulative release rate of 22.85%.

### 3.5 Fitting of drug release profiles

The release of paclitaxel from drug-loaded films involves complex mass transfer processes influenced by multiple factors, including drug solubility, diffusion behavior, pore architecture, and ultrasound-mediated enhancement. Guided by classical dissolution-diffusion theories such as the Noyes–Whitney equation and Fick’s laws, a kinetic model of drug release was established by integrating key variables—type and concentration of hydrophilic modifiers, microporous structure, and ultrasound exposure. Data analysis, regression, and curve fitting were conducted using Origin 2017, with model parameters systematically optimized. The goodness of fit was assessed using the coefficient of determination (*R* ^2^), where higher values indicate closer agreement between the model predictions and experimental results, thereby enabling reliable modeling of release rate over time.

**FIGURE 4:**
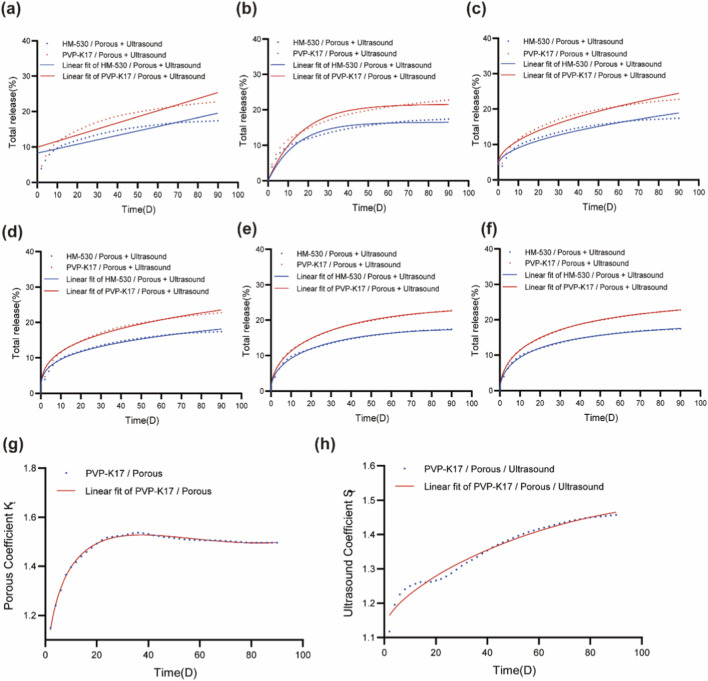
Drug release kinetics of paclitaxel-loaded airway stents with different hydrophilic modifications. **(a)** Cumulative release profile and zero-order model fitting. **(b)** Cumulative release profile and first-order model fitting. **(c)** Cumulative release profile and Higuchi model fitting. **(d)** Cumulative release profile and Ritger-Peppas model fitting. **(e)** Cumulative release profile and diffusion-relaxation model fitting. **(f)** Cumulative release profile and comprehensive model fitting. **(g)** Optimal fitting curve for the pore-formation coefficient (K_t_). **(h)** Optimal fitting curve for the ultrasound-enhancement coefficient (S_t_).

We characterized the release kinetics of the PVP K17/Porous and HM 530/Porous stents under ultrasound by assessing six release models: zero-order, first-order, Higuchi, Ritger-Peppas, diffusion-relaxation, and a comprehensive model. The experimental data were fitted to these models to determine the best fit. The corresponding experimental release curves and their fitted profiles are presented in Figure 4, while the fitted equations and associated *R* ^2^ values are summarized in Table 1. These models were employed to elucidate the underlying release mechanisms and to determine the most appropriate mathematical representation of the drug release behavior for each stent formulation.

**TABLE 1:** Fitting parameters of PTX release kinetics from stents with different hydrophilic modifications.

Hydrophilic modifications	Equation	Adj.R-square	Residual sum of squares	Reduced Chi-Sqr
Zero-order model (y = kt + b)				
HM-530	y = 0.1249 t + 8.2888	0.765	150.668	​
PVP-K17	y = 0.1711 t + 10.0038	0.799	231.465	​
First-order model (y = k(1 - e^-bt^))				
HM-530	y = 16.5380(1-e−^0.0700^ ^t^)	0.943	36.358	0.826
PVP-K17	y = 21.5962(1-e−^0.0613^ ^t^)	0.954	52.834	1.201
Higuchi model (y = kt^1/2^ + b)				
HM-530	y = 1.5514 t^1/2^ + 4.1620	0.927	46.643	1.060
PVP-K17	y = 2.1021 t^1/2^ + 4.4911	0.948	59.906	1.361
Ritger-Peppa model (y = kt^n^ + b)				
HM-530	y = 5.3821 t^0.2777^–0.6251	0.985	9.441	0.220
PVP-K17	y = 6.2489 t^0.3029^ + 0.85152	0.989	12.497	0.291
Diffusion-relaxation model (y = kt^1/2^ + bt + c)				
HM-530	y = 3.3159 t^1/2^–0.1603 t + 0.2893	0.997	2.253	0.052
PVP-K17	y = 4.1290 t^1/2^–0.1842 t + 0.0425	0.999	1.332	0.031
Comprehensive model (y = kt^1/2^ + bt + ct^2^ + d)				
HM-530	y = 3.6984 t^1/2^–0.2277 t+0.0004 t^2^-0.1280	0.997	1.549	0.037
PVP-K17	y = 4.4212 t^1/2^–0.2357 t+0.0003 t^2^-0.2763	0.999	0.922	0.022

As shown in Figure 4, the zero-order model failed to adequately represent the observed release behavior, and both the first-order and Higuchi models also exhibited poor fitting performance, as reflected by their low *R* ^2^ values. The Ritger–Peppas model provided a moderately improved fit, though some deviations persisted. In contrast, the diffusion–relaxation model and the comprehensive model showed the closest agreement with the experimental release profiles. This difference can be attributed to the fact that the first three models assume simplified, single-mechanism release processes, which are insufficient to describe the complex, multi-factor release behavior observed in these functionalized stents. By comparison, the diffusion–relaxation and comprehensive models incorporate multiple contributing mechanisms—such as drug diffusion, polymer relaxation, and structural effects—making them more appropriate for simulating the complex release kinetics of porous and ultrasound-enhanced drug-eluting stents.

Based on the fitting results in Figure 4 and Table 1, the comprehensive model achieved the highest accuracy, with an *R* ^2^ value of 0.999 for the PVP-K17-modified stent, indicating nearly perfect alignment with experimental data. The HM-530-modified stent also showed a strong fit, with an *R* ^2^ of 0.997. The Comprehensive model integrates mathematical components from several classical models and incorporates higher-order polynomial terms, allowing it to more fully represent the complex, multi-mechanistic drug release behavior. Analysis of the comprehensive model equation revealed that the coefficient of the t^1/^ ^2^ term was the largest, suggesting Fickian diffusion is the dominant mechanism during the early release phase. The coefficient of the linear t term was moderate, indicating contributions from polymer relaxation and dissolution processes, while the t^2^ term had the smallest coefficient, implying a limited role of erosion in the overall release process.

The significantly larger coefficient preceding the t^1/^ ^2^ term in the comprehensive model, compared to those of other terms, confirms that diffusion serves as the predominant mechanism governing drug release in the PVP-K17-modified stent, with other processes playing relatively minor roles. Introducing a porous structure into the stent increases its specific surface area, which elevates the diffusion coefficient and further amplifies the magnitude of this linear t^1/^ ^2^-related coefficient. Under ultrasound exposure, cavitation effects further enhance molecular diffusion, resulting in an even greater increase in this coefficient and a correspondingly more pronounced acceleration of the release rate.

The coefficient associated with the linear t term reflects contributions from polymer relaxation or swelling during drug release. In contrast to PS, hydrophilic modifiers such as PVP-K17 possess lower molecular weights and contain hydrophilic functional groups. When immersed in PBS, these modifiers interact strongly with aqueous medium, gradually dissolving from the film and promoting water ingress into the polymer matrix. This leads to more substantial relaxation or swelling in the modified films, which is quantitatively reflected in a larger t-term coefficient.

The coefficient preceding the t^2^ term is negligible—approaching zero—due to the fully cross-linked and cured nature of the outer silicone layer, which undergoes minimal degradation or erosion under physiological conditions. Consequently, erosion-mediated release plays no significant role in the overall drug release process. The observed gradual decline in release rate over time is thus predominantly governed by diffusion and polymer relaxation mechanisms.

### 3.6 Construction of the drug release model for stents

Based on the results in Table 2, the drug release behavior of the stents can be accurately described by the comprehensive model Equation 1:

$$y=A\times t^{1/2}+B\times t+C\times t^{2}+D$$ (1)

where coefficient A corresponds to Fickian diffusion, B reflects contributions from dissolution and polymer relaxation, and C is associated with erosion-mediated release.

**TABLE 2:** Comprehensive model equations for drug release profiles.

Stent types	Equation	Adj. R-Square	Reduced chi-Sqr
**Pure silicone (PS)**	y = 2.0453 t^1/2^–0.1518 t+0.0003 t^2^+0.0814	0.999	0.003
**PS/PEG-600**	y = 2.4175 t^1/2^–0.2247 t+0.0008 t^2^+0.6811	0.986	0.047
**PS/HM-530**	y = 2.0301 t^1/2^–0.1149 t+0.0009 t^2^-0.1312	0.999	0.003
**PS/PVP-K17**	y = 2.7261 t^1/2^–0.2409 t+0.0008 t^2^+0.2758	0.997	0.013
**PS/PVP-K17/Porous**	y = 3.9231 t^1/2^–0.2921 t+0.0006 t^2^-0.2761	0.999	0.011
**PS/PVP-K17/Porous/Ultrasound**	y = 4.4212 t^1/2^–0.2357 t+0.0003 t^2^-0.2763	0.999	0.022

Taking the PVP-K17–modified stent as an example, the original release profile is represented by the comprehensive model Equation 2:

$$y=2.7261t^{1/2}‐0.2409t+0.0008t^{2}+0.2758$$ (2)

This expression served as the baseline for developing extended models that account for the effects of pore formation and ultrasound assistance. Analysis of the fitted coefficients reveals that in the PVP-K17/Porous stent, the absolute values of A and B increased to 3.9231 and 0.2921, respectively, indicating enhanced diffusion and more pronounced relaxation contributions. Meanwhile, the value of C decreased slightly to 0.0006, confirming that erosion continues to play a negligible role even after the introduction of porosity.

#### 3.6.1 Porosity-based drug release model for airway stents

Based on the cumulative release profiles of PVP-K17 and PVP-K17/Porous stents obtained from Figure 3f, a pore-enhancement coefficient (k_t_) was introduced to quantitatively characterize the effect of porosity on drug release. The coefficient k_t_ is defined as the ratio of the cumulative drug release from the PVP-K17/Porous stent to that from the unperforated PVP-K17 stent at the same time point *t*, as expressed in Equation 3:

$$y=\frac{y_{PVP‐K17/Porous}}{y_{PVP‐K17}}$$ (3)

A scatter plot was constructed with release time (*t*) as the independent variable and the corresponding pore-enhancement coefficient (k_t_) values as the dependent variable (Figure 4g). Regression analysis was performed to fit the data, yielding the optimal fitting curve (solid line in Figure 4g) described by Equation 4 with a high coefficient of determination (*R* ^2^ = 0.996):

$$K_{t}=0.2701t^{1/2}‐0.0281t+0.0008t^{2}+0.8142$$ (4)

By incorporating this coefficient into the comprehensive release model, a modified drug release Equation 5 was developed to characterize the release kinetics under porous conditions:

$$y=\left(A\times t^{1/2}+B\times t+C\times t^{2}+D\right)\times K_{t}$$ (5)

The enhancement of drug release rates due to porosity was more evident during the mid-to-late stages of release than in the initial phase. This trend can be explained by the dominant mass-transfer mechanisms operating at different stages. In the early phase, a high concentration of PTX is located on or near the surface of the drug-loaded membrane. Release at this stage is governed mainly by dissolution and diffusion, driven by a steep concentration gradient and relatively high diffusion coefficients. As a result, the contribution of microporous channels formed through porosity remains limited, given the already efficient diffusion across the unmodified matrix surface.

In the mid-to-late stages, however, surface-associated PTX becomes depleted, and drug molecules must diffuse from the inner regions of the membrane to the surface before release. At this point, the interconnected microporous networks created by porosity serve as efficient transport pathways, facilitating the migration of PTX from the interior to the surface. This structural enhancement compensates for the declining concentration gradient and the inherently lower diffusivity in the deeper regions of the membrane. The increased specific surface area and shortened diffusion paths resulting from microporosity collectively enhance drug transport—consistent with Fick’s second law and supported by empirical observations in porous polymer-based delivery systems.

#### 3.6.2 Ultrasound-assisted drug release model for airway stents

Analysis of Table 2 indicates that in the comprehensive release model, coefficient A for the PVP-K17/Porous/Ultrasound stent increased to 4.4212 from 3.9231 for the PVP-K17/Porous stent, reflecting a pronounced enhancement in diffusion-controlled drug release. In contrast, the absolute values of coefficients B and C—associated with relaxation and erosion mechanisms, respectively—decreased from 0.2921 to 0.2357 and from 0.0006 to 0.0003. These results imply that ultrasound assistance predominantly intensifies the diffusion component of drug release, with minimal effect on polymer relaxation or erosion.

Based on the cumulative release profiles in Figure 3f, an ultrasound enhancement factor (S_t_) was defined as the ratio of cumulative release from the PVP-K17/Porous/Ultrasound stent to that from the PVP-K17/Porous stent at identical time points. A scatter plot was generated with release time (t) as the abscissa and S_t_ as the ordinate (Figure 4h). The calculation of S_t_ is given by Equation 6:

$$y=\frac{y_{PVP‐K17/Porous+Ultrasound}}{y_{PVP‐K17/Porous}}$$ (6)

Through regression analysis and curve fitting, the optimal equation characterizing the time-dependent ultrasound enhancement coefficient was obtained. As illustrated in Figure 4h, the fitted curve accurately represents the ultrasound-mediated drug release behavior of the stent, with a high coefficient of determination (*R* ^2^ = 0.977). The resulting fitted Equation 7 is expressed as follows:

$$S_{t}=0.0266t^{1/2}‐0.0021t‐0.0001t^{2}+1.1226$$ (7)

Therefore, the drug release model for stents incorporating both pore-structure enhancement and ultrasound activation can be formulated as Equation 8:

$$y=\left[\left(A\times t^{\frac{1}{2}}+B\times t+C\times t^{2}+D\right)\times K_{t}\right]\times S_{t}$$ (8)

The effect of ultrasound assistance on drug release was relatively modest during the initial 2 days. However, the enhancement factor (S_t_) increased sharply between days 2 and 6, remained elevated through day 20, and continued to rise thereafter. This pattern can be explained by the initial abundance of PTX on the surface of the drug-loaded membrane, where release is dominated by dissolution and diffusion. During this early phase, ultrasound-induced cavitation contributes minimally to overall release. As the surface PTX depletes in later stages, drug release becomes increasingly dependent on the migration of molecules from the inner regions of the membrane to the surface. Ultrasound-generated cavitation microbubbles collapse and produce localized shear forces that disrupt drug–matrix interactions and promote drug transport toward the surface. Consequently, ultrasound exerts a more pronounced enhancement effect in the mid-to-late release phases, as reflected in the sustained increase in S_t_ beyond day 20.

In contrast, as shown in Figure 4g, the pore-enhancement coefficient (K_t_) begins to decline after day 30, whereas the ultrasound enhancement factor (S_t_) continues to increase. This indicates that the release-enhancing effect produced by ultrasound-induced cavitation exceeds the contribution of microchannels formed through porosity during long-term drug elution.

### 3.7 *In vitro* cell experiments with stent

To assess the cytocompatibility of the modified silicone material and to quantitatively evaluate the differential biological effects of paclitaxel under release-relevant conditions, four representative cell types were tested, including L929 mouse fibroblasts, HFL-1 human lung fibroblasts, BEAS-2B human bronchial epithelial cells, and HBE135 primary human bronchial epithelial cells. Extracts of the PVP-K17-modified silicone were prepared according to ISO 10993–12, and cytotoxicity was interpreted according to ISO 10993–5. In addition, a PTX treatment medium was prepared in the extract medium of the drug-free PVP-K17-modified silicone material, using the average PTX release concentration during the first 10 days as the representative exposure level (9.3 μg/mL).

**FIGURE 5:**
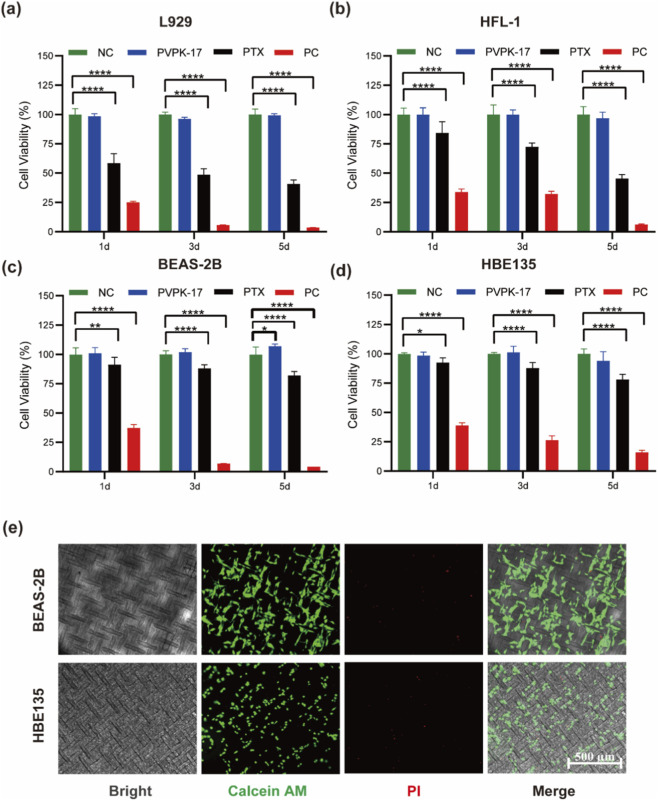
Quantitative evaluation of cell viability under material-extract and PTX treatment conditions. **(a–d)** Viability of **(a)** L929, **(b)** HFL-1, **(c)** BEAS-2B, and **(d)** HBE135 cells after 1, 3, and 5 days of treatment with NC (negative control), PVP-K17 (100% extract of the PVP-K17-modified silicone material), PTX (PTX treatment medium prepared in the extract medium of the drug-free PVP-K17-modified silicone material, based on the average PTX release concentration during the first 10 days of the optimized stent release profile, 9.3 μg/mL), and PC (positive control). Data are presented as mean ± SD (n = 6). *P < 0.05, **P < 0.01, ****P < 0.0001. **(e)** Live-dead staining of HBE135 and BEAS-2B bronchial epithelial cells cultured directly on 5% PVP-K17 silicone films. Scale bar: 500 μm.

Cell viability was quantified by MTT assay after 1, 3, and 5 days of treatment (Figure 5). Across all 4 cell lines, the PVP-K17 group showed minimal cytotoxicity and remained comparable to the negative control (NC). According to ISO 10993–5, a reduction in cell viability by more than 30% is considered cytotoxic, indicating that materials with cell viability ≥70% relative to the negative control are regarded as non-cytotoxic. All viability values in the PVP-K17 group remained above this threshold, indicating that the PVP-K17-modified material exhibited no detectable *in vitro* cytotoxicity. In contrast, the PTX group showed a clear time-dependent reduction in fibroblast viability, particularly in L929 and HFL-1 cells, whereas BEAS-2B and HBE135 epithelial cells retained relatively high viability throughout the same treatment period. The positive control (PC) induced marked cytotoxicity in all cell lines, confirming the sensitivity of the assay system. To further assess fibrosis-related molecular responses in fibroblasts, qPCR analysis was performed in HFL-1 cells under Ctr, TGF-β1, and TGF-β1 + PTX treatment conditions. As shown in Supplementary Figure S2a, TGF-β1 markedly increased the mRNA expression of α-SMA, FIBRO, and COL1, whereas PTX significantly attenuated the induction of all three markers. These results suggest that PTX not only reduces fibroblast viability, but also attenuates TGF-β1-induced profibrotic gene expression in fibroblasts.

To further evaluate the cytocompatibility of the material with respiratory epithelial cells, BEAS-2B and HBE135 cells were seeded directly onto the material and cultured under standard conditions (37 °C, 5% CO_2_). Live-dead staining revealed robust green fluorescence in both cell types (Figure 5e), indicating a high density of viable cells. Only sparse red fluorescence was observed, suggesting minimal cell death. Both BEAS-2B and HBE135 cells retained their typical epithelial morphology—polygonal shape for BEAS-2B and cobblestone-like organization for HBE135—confirming that the material supports epithelial adhesion, proliferation, and viability without inducing detectable cytotoxicity.

To investigate the cell-type-specific effects of PTX, live-dead staining was performed in HFL-1 and HBE135 cells under the PTX treatment condition defined above.

**FIGURE 6:**
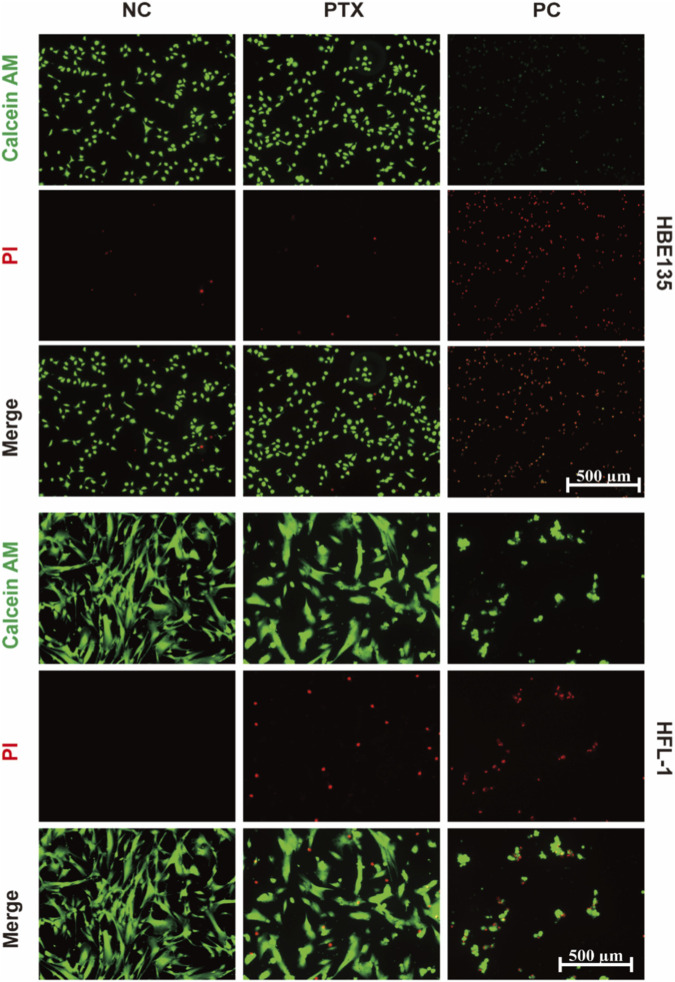
Live-dead staining of HBE135 and HFL-1 cells after treatment with PTX-treatment medium (PTX), negative control (NC), and positive control (PC). Scale bar: 500 μm.

As shown in Figure 6, HBE135 cells in the PTX-treated group displayed dense green fluorescence comparable to the NC, with only sporadic red signals, indicating negligible cytotoxicity. In contrast, HFL-1 cells exposed to PTX showed a marked increase in red-stained dead cells relative to the NC, although viable (green) cells still constituted the majority. Critically, the level of cell death observed in HFL-1 cells remained substantially lower than that in the PC group.

These results demonstrate that PTX under the tested treatment condition induced greater injury in HFL-1 fibroblasts while sparing bronchial epithelial cells supporting the potential of PTX-eluting stents to modulate airway fibrotic responses through preferential targeting of fibroblasts.

**FIGURE 7:**
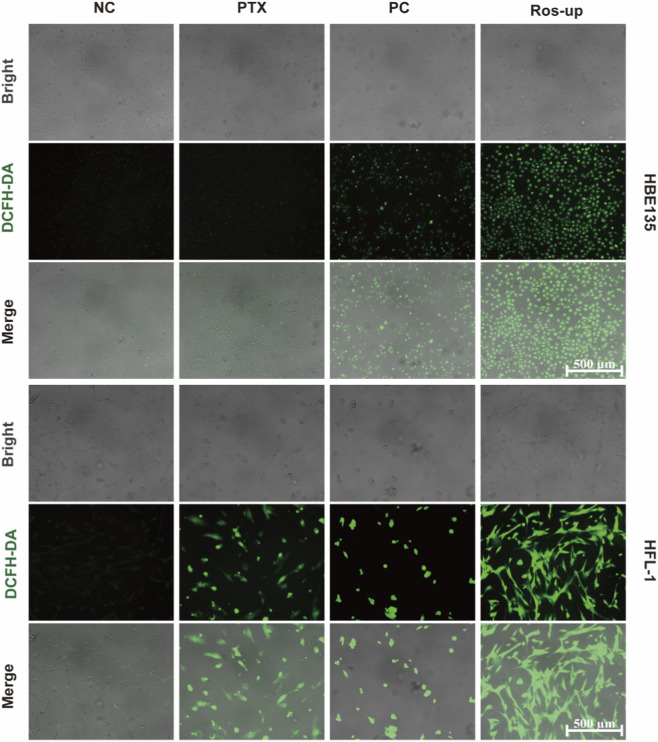
Intracellular ROS levels in HBE135 and HFL-1 cells following treatment with PTX-treatment medium (PTX), negative control (NC), and positive control (PC). Scale bar: 500 μm.

To elucidate the mechanism underlying the cell-specific cytotoxicity of PTX, we measured intracellular reactive oxygen species (ROS) levels in HBE135 and HFL-1 cells using DCFH-DA staining (green fluorescence indicates ROS). As shown in Figure 7, PTX-treated HBE135 cells exhibited minimal green fluorescence, similar to the NC group, indicating that PTX did not significantly induce ROS generation in this epithelial cell type. In contrast, HFL-1 fibroblasts exposed to PTX showed markedly enhanced green fluorescence compared to the NC group, though the intensity remained lower than that in the PC or ROS-upregulated groups.

This differential ROS response aligns with previous live-dead staining results: epithelial cells, with low ROS induction, maintained high viability, whereas fibroblasts, under elevated ROS, experienced more substantial damage. Given the established role of ROS as key mediators of cellular injury, the distinct sensitivity of these cell types to oxidative stress suggests the selective toxicity of PTX—fibroblasts appear more vulnerable to ROS-mediated damage, while epithelial cells remain relatively resistant. This mechanistic interpretation further supports the selective anti-fibroproliferative effect of PTX while indicating relative preservation of airway epithelial cells.

## 4 Discussion

This study presents a silicone-based long-acting paclitaxel-eluting airway stent and systematically explores strategies to enhance drug release while maintaining biocompatibility and mechanical performance. The developed stent addresses a critical clinical challenge in airway intervention: restenosis resulting from chronic inflammatory responses post-stenting. Our integrated strategy combining hydrophilic modification, pore architecture engineering, and ultrasound activation enabled optimized paclitaxel release over a 90-day period. The results show that through precise regulation of formulation and processing parameters—including the incorporation of PEG-600, PVP-K17, and HM-530 modifiers—both the drug release rate and cumulative delivery were significantly enhanced, reaching a maximum cumulative release of 22.85%. This represents an approximately threefold improvement compared to unmodified silicone matrices.

Although the optimized stent contained 24 mg paclitaxel, this nominal amount should be interpreted as a reservoir loading rather than as the free drug concentration expected in the airway lumen or intramural tissue. Paclitaxel has been shown to inhibit human pulmonary fibroblast proliferation in a dose- and time-dependent manner *in vitro* (Wang et al., 2010), and local airway application has also demonstrated clinical activity as an adjuvant treatment for benign cicatricial airway stenosis with a high durable remission rate and few reported complications (Qiu et al., 2016). In addition, a previous paclitaxel-loaded tracheal stent study reported a drug loading of approximately 16.38 mg/stent and a sustained release rate of 0.3763 mg/day, which that study described as a therapeutic level (Kong et al., 2014). In our system, the optimized formulation achieved a cumulative release of 22.85% over 90 days, indicating that only a fraction of the total payload was gradually liberated during the tested window, whereas the remaining drug likely remained in deeper domains of the hydrophobic silicone matrix as a slowly diffusible reservoir, consistent with prior stent-coating studies showing that polymer composition and structure can strongly modulate controlled release behavior (Guo et al., 2009). However, the present 90-day *in vitro* data do not determine whether this retained fraction would continue to be released only very slowly over prolonged implantation or whether a portion may remain effectively trapped within the silicone matrix. Consistent with this interpretation, a supplementary post-90-day release test showed no measurable additional PTX release after the optimized stent was reintroduced into fresh release medium under the same *in vitro* release conditions (Supplementary Figure S1a), suggesting that PTX release had approached a plateau by this time point. From a translational perspective, the rationale for selecting a 24 mg loading was therefore not to generate a high bulk luminal concentration, but to maintain prolonged local exposure at the stent–tissue interface despite the inherently diffusion-limited release behavior of silicone-based carriers. This interpretation is further supported by *in vivo* evidence showing that paclitaxel-eluting tracheal stents can reduce granulation tissue formation while maintaining acceptable safety profiles in a canine model (Wang et al., 2016). Because paclitaxel is highly hydrophobic and poorly water-soluble, PBS containing 1% SDS was used here to maintain sink conditions for *in vitro* release quantification rather than to reproduce the full biochemical environment of airway lining fluid (Gupta et al., 2014). Accordingly, the actual intramural exposure achieved *in vivo* may still be influenced by mucus barriers and mucociliary clearance, and should be further defined in future pharmacokinetic and animal studies (Pangeni et al., 2023).

Although ultrasound substantially enhanced cumulative PTX release *in vitro*, the present stimulation settings (40 kHz, 180 W, 15 min every 24 h) should be interpreted as a proof-of-concept external triggering regimen rather than a finalized clinical protocol. Prior reviews of ultrasound-mediated drug delivery have shown that acoustic cavitation, microstreaming, and related mechanical bioeffects can enhance mass transport and disrupt drug–matrix interactions, while also emphasizing the need to consider thermal and safety effects *in vivo* (Wu and Nyborg, 2008; Wei et al., 2021; Zhang et al., 2022). However, translation of this strategy to the airway is not straightforward, because ultrasound waves reflect strongly at air–tissue interfaces and do not propagate effectively through air, making acoustic coupling in the air-containing airway fundamentally different from the liquid-immersion release system used in the present study (Dabo-Trubelja, 2023; Lin et al., 2023). A clinically relevant implementation would therefore likely require a locally coupled delivery strategy rather than direct propagation through the intraluminal air column. In practice, this may require positioning the ultrasound source in close approximation to the airway wall or using a fluid-coupled interface to improve local energy transfer to the stented segment. Accordingly, the current *in vitro* ultrasound parameters should not be directly extrapolated to clinical airway intervention. Instead, future translation will require dedicated optimization of acoustic coupling, delivery geometry, and exposure safety under airway-specific conditions. As supportive evidence for short-term material stability under the tested post-release ultrasound conditions, supplementary SEM, FTIR, and TGA analyses indicated that the silicone-based matrix retained basic structural and physicochemical integrity without obvious microstructural deterioration (Supplementary Figures S1b-d). Consistently, supplementary radial compression testing showed that the radial support force–compression curves remained broadly comparable after prolonged release and after an additional 3 days of ultrasound exposure, suggesting preservation of mechanical support performance over the tested therapeutic window (Supplementary Figure S1e). Given that novel airway stent platforms remain in an early stage of translational development, such evaluation should include clinically compatible delivery strategies as well as assessment of local mucosal tolerance and repeated-exposure safety before *in vivo* application (Bashour and Lazarus, 2024; Tian et al., 2022).

From a materials science perspective, silicone—an inert polymer widely used in airway support applications—demonstrates excellent biocompatibility, design flexibility, and mechanical stability. However, its intrinsic hydrophobicity, dense structure, and non-degradable nature impose inherent limitations for drug delivery applications. In this study, the implementation of a porous structural design substantially increased the material’s specific surface area and created continuous diffusion pathways, thereby promoting drug transport toward the stent surface. This approach aligns with the findings of Wang et al., who reported that fenestrated silicone stents not only exhibited enhanced biomechanical and drug release performance compared to conventional solid designs but also reduced localized tissue stress concentration and mucosal injury post-implantation (Wang et al., 2025).In parallel, the application of a hydrophilic-modified coating improved not only surface wettability but also interfacial hydrophilicity and molecular diffusivity, synergistically enhancing drug release in aqueous physiological environments. Corroborating this strategy, Cho developed a hydrophilic polymer-coated airway stent that demonstrated reduced mucus adhesion and minimized tissue damage in animal models, indicating enhanced biotolerance and therapeutic safety within the airway microenvironment (Cho et al., 2024). The marked increase in drug release flux observed in our experimental results further validates the functional effectiveness of these material modifications (Figure 3e).

The predominance of the t^1^/^2^ term in the kinetic model suggests that drug release was primarily diffusion-controlled under the present static *in vitro* conditions. However, the airway is a mechanically and hydrodynamically dynamic environment. Airflow-dependent shear, cyclic respiratory deformation, cough-related transient compression, and mucus transport may alter boundary-layer thickness, matrix hydration, and effective diffusion path length over time, thereby changing the relative contributions of diffusion and matrix relaxation during long-term release. Accordingly, the current kinetic interpretation should be viewed as a simplified baseline derived from an immersion-based release system rather than a full surrogate for the *in vivo* airway milieu. In addition, the airflow consequence of the present stent geometry was not directly measured in this study, but a basic geometric analysis can still provide quantitative context. For the present design, the outer diameter is 16 mm, the wall thickness is 1.5 mm, the nominal inner diameter is 13 mm, and the stent length is 80 mm. This corresponds to a luminal cross-sectional area of approximately 132.7 mm^2^. If the wall thickness were reduced from 1.5 mm to 1.0 mm while maintaining the same outer diameter, the nominal inner diameter would increase from 13 mm to 14 mm, and the luminal cross-sectional area would increase from approximately 132.7 mm^2^–153.9 mm^2^ (about 16.0%), illustrating that even a modest change in wall thickness can materially affect the effective lumen available for airflow. However, the present device was not designed to maximize airflow alone. Rather, the 1.5 mm wall thickness was considered a practical compromise to maintain structural support and to accommodate the drug-eluting multilayer architecture required for sustained local delivery. Thus, the current geometry should be interpreted as a balanced design rather than as an airflow-optimized configuration. Although this simplified comparison does not replace patient-specific aerodynamic analysis, it provides a basic quantitative context showing that the airflow consequence of the design should not be assumed to be negligible. Prior computational analyses have likewise shown that stent placement can alter pressure drop and flow distribution in a geometry-dependent manner (Ho et al., 2012). Therefore, future work should integrate computational fluid dynamics, *ex vivo* flow testing, or direct comparison with established commercial silicone stents to better relate device geometry to airflow resistance and drug-transport behavior (Johnson et al., 2022; Lin et al., 2023; Ratnovsky et al., 2015). Airway-stent development remains highly dependent on careful patient- and stent-specific selection, and in future clinical application, stent sizing should be selected according to the target airway diameter and patient-specific anatomy so as to minimize any adverse impact on airflow and ventilation.

Nevertheless, the cumulative drug release from this system (22.85%) remains lower than that of many reported biodegradable platforms. For instance, Guo et al. achieved nearly 90% release within 90 days using hybrid polyurethane stent coatings, a result attributed to the polymer’s controlled hydrolytic degradation and tunable hydrophilicity (Guo et al., 2009). Despite this limitation, silicone stents retain unique clinical advantages such as excellent shape retention, mechanical reliability, and removability. These properties make them particularly suitable for benign central airway stenosis and other reversible conditions requiring temporary long-term support.

Our *in vitro* results consistently indicate that the optimized stent formulation combines favorable material cytocompatibility with selective anti-fibroproliferative activity. As shown in Figure 5, the PVP-K17-modified silicone extract remained non-cytotoxic in both fibroblast and epithelial cell models, with all viability values exceeding the 70% threshold defined by ISO 10993–5, confirming the biosafety of the modified matrix itself. By contrast, under the PTX treatment condition, fibroblast viability decreased in a time-dependent manner, whereas bronchial epithelial cells remained relatively well preserved (Figure 5). These quantitative findings were further supported by the live-dead staining results, in which PTX treatment caused minimal death in epithelial cultures but substantially greater injury in fibroblast cultures (Figure 6). Together, these data support the view that PTX preferentially inhibits fibroblasts while relatively sparing the regenerative airway epithelium, thereby providing a potentially favorable therapeutic basis for limiting fibroproliferative restenosis after stent implantation. In addition, qPCR analysis showed that PTX attenuated TGF-β1-induced upregulation of α-SMA, FIBRO, and COL1 in HFL-1 cells, providing additional molecular support that PTX counteracts profibrotic transcriptional responses in fibroblasts (Supplementary Figure S2a). A scratch wound-healing assay showed that BEAS-2B cells retained substantial migratory repair capacity under PTX exposure, further supporting relative preservation of epithelial healing-related function under the tested conditions (Supplementary Figure S2b).

A plausible explanation for this selective cellular response is that the cytotoxic effect of PTX in our system is at least partly redox-mediated, as suggested by the intracellular ROS results shown in Figure 7. In cancer models, paclitaxel can elevate ROS to cytotoxic levels in susceptible tumor cells while relatively sparing non-malignant cells with intact redox homeostasis (Liu and Wang, 2015). In the airway context, epithelial antioxidant defenses (e.g., SOD and catalase) are well documented and catalytic augmentation mitigates oxidative injury *in vivo*, which may contribute to the relative protection observed in bronchial epithelia (Ansar et al., 2020; Kinnula and Crapo, 2003). Fibrotic fibroblasts reside in a state of chronic oxidative stress that sustains their activated/myofibroblast phenotype and reflects NOX-like ROS dependence (Bocchino et al., 2010). Within the fibrotic niche, ROS and TGF-β engage in feed-forward/feedback circuits that amplify profibrotic signaling and drive excess extracellular-matrix accumulation, intensifying redox sensitivity (Richter and Kietzmann, 2016). Therefore, our results are best explained by PTX exacerbating ROS in these already-stressed fibroblasts, surpassing a critical threshold for survival, while epithelial cells effectively buffer the insult.

Nevertheless, further validation of this mechanism is imperative. Future work should directly probe ROS-dependent pathways by assessing key indicators such as mitochondrial membrane potential (ΔΨm), the intracellular GSH/GSSG ratio, and the expression of NOX4 and other NADPH oxidase subunits. Crucially, the central role of oxidative stress should be confirmed by testing whether antioxidant pre-treatment (e.g., with N-acetylcysteine) attenuates PTX-induced fibroblast cytotoxicity. Beyond *in vitro* studies, *in vivo* validation using relevant animal models of airway stenosis is essential to evaluate the long-term therapeutic efficacy, tissue remodeling dynamics, epithelial regeneration, and overall biocompatibility of the PTX-eluting stent under physiological conditions. Collectively, these investigations will provide critical translational insights necessary for optimizing stent design and advancing this therapeutic strategy toward clinical application in fibroproliferative airway diseases.

Finally, the present study should be interpreted as an *in vitro* proof-of-concept focused on formulation optimization, release behavior, and initial cell-type selectivity. Although the epithelial and fibroblast assays used here provide a useful first-pass evaluation of cytocompatibility and selective anti-fibroproliferative activity, they do not fully recapitulate the granulation-prone airway microenvironment, which also involves epithelial remodeling, immune-cell recruitment, cytokine signaling, mucus responses, and extracellular-matrix reorganization (Henderson et al., 2020; Johnson et al., 2022; Li et al., 2025; Liu et al., 2021). More physiologically relevant co-culture, barrier-function, inflammatory, and air–liquid interface models will therefore be needed in future studies to strengthen translational interpretation. In addition, successful clinical translation of a PTX-eluting silicone airway stent will depend not only on release performance, but also on manufacturability, batch-to-batch consistency, shelf stability, and sterilization compatibility. Because commonly used sterilization approaches may affect drug stability, polymer structure, and release behavior, these issues should be systematically evaluated during subsequent device-development studies (Liu et al., 2020; Tian et al., 2022; Xu et al., 2020).

## Data Availability

The original contributions presented in the study are included in the article/Supplementary Material, further inquiries can be directed to the corresponding authors.
