# Supplementary material for: Paclitaxel-eluting silicone airway stent with sustained drug release and potent anti-fibrotic activity
Source: Front Bioeng Biotechnol. 2026 May 12;14:1805822. doi: 10.3389/fbioe.2026.1805822 (PMC13201427; doi:10.3389/fbioe.2026.1805822)
Supplement: Supplementary file 1 [file Supplementaryfile1.docx]

Supplementary Material

# Supplementary Methods

**1.1 Fourier transform infrared spectroscopy (FTIR)**

Fourier transform infrared spectroscopy (FTIR) was performed using a Fourier transform infrared spectrometer (TENSOR 27, Bruker, Germany) to assess the chemical structure features of the samples. The samples were prepared using the KBr pellet method at a sample-to-KBr mass ratio of 1:100. Spectra were recorded over the range of 4000–400 cm^⁻¹^. The resulting spectra were analyzed by comparing the characteristic absorption bands of the different silicone-based samples.

**1.2 Thermogravimetric analysis (TGA)**

Thermogravimetric analysis (TGA) was performed using a thermogravimetric analyzer (TGA550, TA Instruments, USA) to evaluate the thermal stability and weight-loss behavior of the samples. Approximately 10 mg of silicone airway stent sample or related constituent material was placed in a crucible and heated from 20 °C to 800 °C at a rate of 5 °C/min under a nitrogen atmosphere. The sample mass was continuously recorded as a function of temperature, and the resulting TG curves were used to compare the thermal decomposition and weight-loss characteristics of the different samples.

**1.3 RT-qPCR assay**

HFL-1 cells were seeded into 6-well plates at a density of 1 × 10^5^ cells per well. After adherence, the cells were cultured in medium containing 1% FBS for 24 h for serum starvation, followed by stimulation with 10 ng/mL TGF-β1 (MedChemExpress, MCE) for 24 h. For PTX treatment, cells were co-treated with 10 ng/mL TGF-β1 and PTX (9.3 μg/mL) for 24 h. Total RNA was then extracted using RNAiso Plus reagent (Takara Biotechnology, Dalian, China; #9108) according to the manufacturer’s instructions. The purity and concentration of RNA were assessed by measuring the A260/A280 ratio. cDNA was synthesized using PrimeScript RT Master Mix (Takara, Dalian, China; #RR036A) at 37°C for 15 min and 85°C for 5 s. Quantitative PCR was carried out using TB Green Premix Ex Taq II (Takara, Dalian, China; #RR820A) on a real-time PCR system. GAPDH served as the internal reference, and relative gene expression was analyzed using the 2^-ΔΔCt method.

**1.4 Scratch wound-healing assay**

Cell migratory capacity was evaluated using an ibidi 3-well culture-insert wound-healing model. BEAS-2B cells were suspended at a density of 1 × 10^5^ cells/mL, and 80 μL of cell suspension was added into each chamber of the insert to generate two defined cell-free gaps. After incubation at 37 °C for 24 h, the insert was gently removed to create the wound areas. Fresh medium containing NC, PVP-K17 extract, PTX treatment medium (drug-free PVP-K17-modified silicone extract containing 9.3 μg/mL paclitaxel), or PC medium was then added according to the experimental grouping and wound closure was imaged at 0, 48, and 96 h using an inverted microscope (Axio Observer 5, ZEISS, Germany).

# Supplementary Figures and Tables

## Supplementary Figures


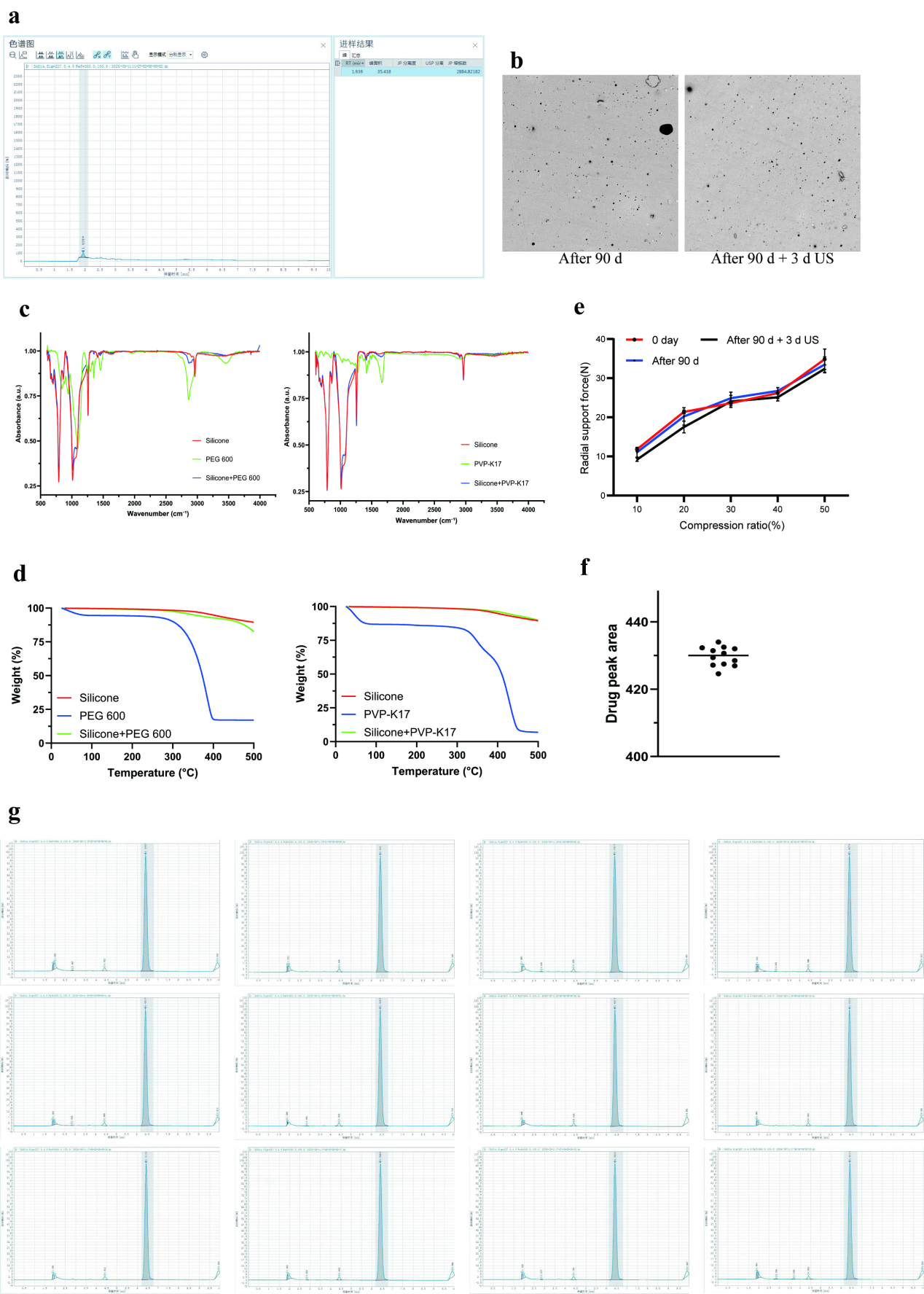


**Supplementary Figure 1.** (a) HPLC analysis of PTX after the optimized stent was reintroduced into fresh release medium following completion of the 90-day release study under the same in vitro release conditions. (b) SEM images of the optimized stent after completion of the 90-day release study (After 90d) and after an additional 3 days of ultrasound exposure (After 90d + 3d ultrasound). (c) FTIR spectra. Left: silicone, PEG600, and the PEG600-containing silicone stent after completion of the 90-day release study followed by an additional 3 days of ultrasound exposure. Right: silicone, PVP-K17, and the optimized stent after an additional 3 days of ultrasound exposure. (d) TGA curves. Left: silicone, PEG600, and the PEG600-containing silicone stent after completion of the 90-day release study followed by an additional 3 days of ultrasound exposure. Right: silicone, PVP-K17, and the optimized stent after an additional 3 days of ultrasound exposure. (e) Radial support force–compression ratio curves of the optimized stent at 0 day, after completion of the 90-day release study, and after completion of the 90-day release study followed by an additional 3 days of ultrasound exposure. (f) Statistical comparison of PTX peak areas in release samples from the 12 stent segments. (g) Representative HPLC chromatograms of release samples obtained from 12 stent segments generated by dividing the stent into upper, middle, and lower portions and further subdividing each portion into four equal sectors.


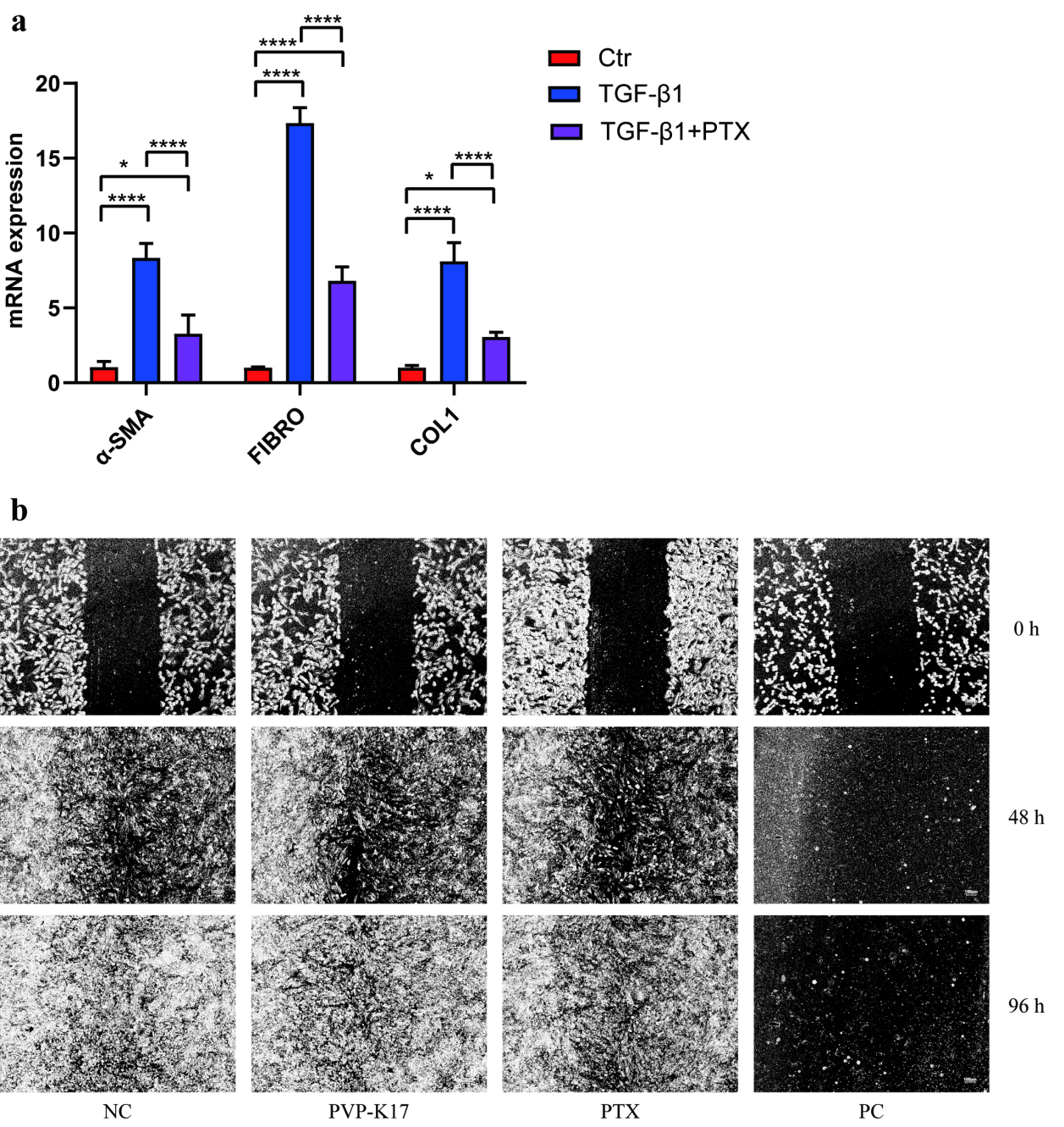


**Supplementary Figure 2.** (a) qPCR analysis of fibrosis-associated markers in HFL-1 cells under TGF-β1 and PTX treatment. Relative mRNA expression of α-SMA, FIBRO, and COL1 in HFL-1 cells under Ctr, TGF-β1, and TGF-β1 + PTX conditions. Data are presented as mean ± SD. Statistical analysis was performed using two-way ANOVA followed by multiple-comparison testing. **P < 0.05, ****P < 0.0001*. (b) Scratch wound-healing assay of BEAS-2B cells under NC, PVP-K17, PTX, and PC conditions.

## Supplementary Tables

Table S1 Drug release profiles of airway stents modified with different hydrophilic agents

| **Stent Type** | **Pure Silicone** | **PEG-600** | **HM-530** | **PVP-K17** |
| --- | --- | --- | --- | --- |
| **Release Rate for the First Two Days (%)** | 2.69 | 4.45 | 2.35 | 3.70 |
| **Day 10 Release Rate (%)** | 0.42 | 0.26 | 0.50 | 0.37 |
| **Day 30 Release Rate (%)** | 0.11 | 0.12 | 0.16 | 0.13 |
| **Day 60 Release Rate (%)** | 0.03 | 0.07 | 0.04 | 0.06 |
| **Day 90 Release Rate (%)** | 0.02 | 0.04 | 0.03 | 0.04 |
| **90-Day Cumulative Release (%)** | 8.34 | 10.00 | 9.57 | 10.48 |

Table S2 Comparison of drug release from different hydrophilic modified stents after pore formation

| **Stent Type** | **HM-530** | **PVP-K17** | **HM-530/Porous** | **PVP-K17/Porous** |
| --- | --- | --- | --- | --- |
| **Release Rate for the First Two Days (%)** | 2.35 | 3.70 | 3.74 | 4.25 |
| **Day 10 Release Rate (%)** | 0.50 | 0.37 | 0.36 | 0.71 |
| **Day 30 Release Rate (%)** | 0.16 | 0.13 | 0.05 | 0.21 |
| **Day 60 Release Rate (%)** | 0.04 | 0.06 | 0.03 | 0.08 |
| **Day 90 Release Rate (%)** | 0.03 | 0.04 | 0.02 | 0.06 |
| **90-Day Cumulative Release (%)** | 9.57 | 10.48 | 9.39 | 15.68 |

Table S3 Comparison of drug release from different hydrophilic modified stents after ultrasound treatment

| **Stent Type** | **HM-530/Porous** | **PVP-K17/Porous** | **HM-530/Porous**  **/Ultrasound** | **PVP-K17/Porous/Ultrasound** |
| --- | --- | --- | --- | --- |
| **Release Rate for the First Two Days (%)** | 3.74 | 4.25 | 3.9 | 4.75 |
| **Day 10 Release Rate (%)** | 0.36 | 0.71 | 0.80 | 0.97 |
| **Day 30 Release Rate (%)** | 0.05 | 0.21 | 0.30 | 0.42 |
| **Day 60 Release Rate (%)** | 0.03 | 0.08 | 0.14 | 0.18 |
| **Day 90 Release Rate (%)** | 0.02 | 0.06 | 0.05 | 0.11 |
| **90-Day Cumulative Release (%)** | 9.39 | 15.68 | 17.42 | 22.85 |
